# Adaptive effect of sericin on hepatic mitochondrial conformation through its regulation of apoptosis, autophagy and energy maintenance: a proteomics approach

**DOI:** 10.1038/s41598-018-33372-4

**Published:** 2018-10-08

**Authors:** Sumate Ampawong, Duangnate Isarangkul, Onrapak Reamtong, Pornanong Aramwit

**Affiliations:** 1 Department of Tropical Pathology, Faculty of Tropical Medicine, Mahidol University, Ratchawithi Road, Ratchathewi, Bangkok, 10400 Thailand; 2 Department of Microbiology, Faculty of Science, Mahidol University, 272, Rama VI Road, Ratchathewi, Bangkok, 10400 Thailand; 3 Department of Molecular Tropical Medicine and Genetic, Faculty of Tropical Medicine, Mahidol University, Ratchawithi Road, Ratchathewi, Bangkok, 10400 Thailand; 4 Bioactive Resources for Innovative Clinical Applications Research Unit and Department of Pharmacy Practice, Faculty of Pharmaceutical Sciences, Chulalongkorn University, PhayaThai Road, Phatumwan, Bangkok, 10330 Thailand

**Keywords:** Sericin, Dimethylglycine Dehydrogenase (DMGDH), Sarcosine Dehydrogenase (SARDH), Mitochondrial Architecture, Prohibitin (PHB2), Preclinical research, Immunopathogenesis

## Abstract

We recently demonstrated that in addition to its protective effect on pancreatic and adrenal biosynthesis, antioxidant properties of sericin decrease blood cholesterol levels and improve the liver mitochondrial architecture. However, little is known about the detailed mechanisms underlying these effects. Using proteomics and electron microscopy, we identified mitochondrial proteins that play important roles in the preservation of the mitochondrial ultrastructure and cholesterol-lowering properties of sericin. Our results showed that sericin maintains the mitochondrial architecture during conditions of high blood cholesterol by regulating apoptotic (NADH-ubiquinone oxidoreductase 75 kDa subunit) and autophagic (mitochondrial elongation factor Tu and prohibitin-2) proteins as well as energy maintenance proteins [haloacid dehalogenase-like hydrolase domain-containing protein 3, succinate dehydrogenase (ubiquinone) flavoprotein subunit, ATP synthase-α subunit precursor, enoyl-CoA hydratase domain-containing protein 3 and electron transfer flavoprotein subunit-α]. Sericin also exerts anti-oxidative properties via aconitate hydratase and Chain A, crystal structure of rat carnitine palmitoyltrasferase 2 proteins. Together, these activities may reduce hepatocytic triglyceride deposition, thereby decreasing steatosis, as demonstrated by the modulatory effects on ornithine aminotransferase, mitochondrial aspartate aminotransferase, acyl-CoA synthase, hydroxyacyl-CoA dehydrogenase and D-beta-hydroxybutyrate dehydrogenase. Sericin activity further balanced nitrogenous waste detoxification, characterised by carbamoyl-phosphate synthase (ammonia), aldehyde dehydrogenase and uricase, or folate biosynthesis via sarcosine dehydrogenase and dimethyl glycine dehydrogenase. These results suggest that sericin maintains the hepatic mitochondrial architecture through apoptotic, autophagic, energy maintenance and anti-oxidative mitochondrial proteins for alleviating hepatic steatosis and promoting liver function under conditions of hypercholesterolaemia.

## Introduction

The blood cholesterol-lowering activity of sericin has been studied for decades^1–5^. Several mechanisms, mainly focussing on competitive effects on cholesterol absorption, retardation of lipidogenesis in the liver and anti-oxidative properties, have been postulated. Our recent studies^1,2^ have demonstrated that sericin improves dyslipidaemia and liver or heart mitochondrial architecture via its anti-oxidative activity, as characterised by the reduction in mitochondrial reactive oxygen species, downregulation of malondialdedhyde (MDA) and upregulation of nuclear factor erythroid 2-related factor (NRF-2). In addition, sericin increases lipase activity by enhancing pancreatic and adrenal gland biosynthesis via aquaporin-1 and tubulib-4β proteins. These effects are associated with the retardation of fat anabolism, resulting in an increased hypocholesterolaemic capacity of sericin, particularly lowering blood cholesterol levels and increasing high-density liproprotein (HDL) levels. In addition, the mitochondria conformation-promoting effect of sericin in the liver and heart is enrolled by fission and fusion phenomena that regulate early-stage apoptosis and maintain mitochondrial morphology and function, respectively^6–11^. As described in our previous studies, sericin downregulates the fission protein dynamin-related protein 1 (DRP1) and upregulates the fusion protein anti-optic atrophy 1 (OPA1). However, precise mechanisms underlying the improvement of dysmorphic mitochondria in the liver and severity of hepatic steatosis in hypercholesterolaemia remain unclear.

Using a hypercholesterolaemia rat model, proteomics and electron microscopy analyses were performed for elucidating mechanisms underlying beneficial effects of sericin that help in treating hypercholesterolaemia. Expression of several proteins in the liver mitochondria and their functions were examined in relation to their fine morphology at any stage of mitochondrial degeneration. The present study provides a better understanding of the pathogenesis of hypercholesterolaemia-induced hepatic steatosis, focussing on mitochondrial functions under the modulatory effects of sericin.

## Results

### Histopathology, blood lipid profiles and mitochondrial conformation

**Figure 1:**
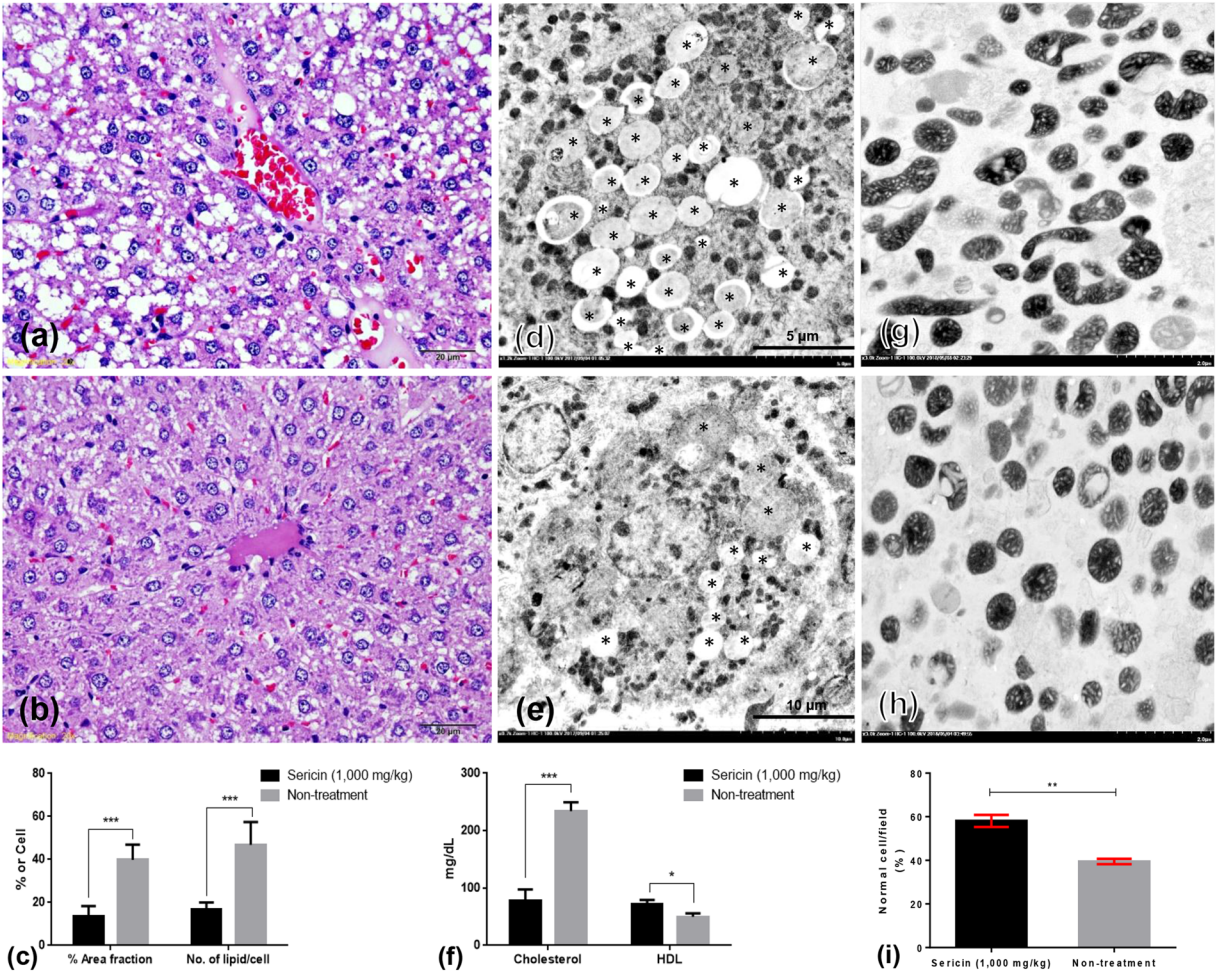
Histopathological changes, blood lipid profiles and mitochondrial ultrastructure in sericin-treated and non-treated rats. The severity of hepatic steatosis in non-treated (**a**,**d**) or sericin-treated (**b**,**e**) high-cholesterol-fed rats was compared and represented by percentage area fraction or number of lipid/cell, respectively (**c**,**f**). Compared with sericin-treated rats, micro- and macrovesicular steatosis were highly presented in the periportal area of non-treated rats (H&E staining). Electron microscopy demonstrated that lipid accumulation (*) in hepatocytes in non-treated rats (**d**) was higher than that in sericin-treated rats (**e**). The bar graph indicates blood cholesterol and HDL levels in sericin-treated and non-treated rats with hypercholesterolaemic; data is represented by mean ± SEM; *p < 0.05, **p < 0.01, and ***p < 0.001 (**f**). Comparison of electron micrograph of the mitochondria architecture between sericin-treated (**g**) and non-treated (**h**) rats.

Histopathology and electron microscopy analyses demonstrated that after ingestion of sericin for 4 weeks, treated rats had lower hepatocyte fat deposition than non-treated rats had (Fig. 1a–e). Sericin also decreased blood cholesterol levels and increased HDL levels (Fig. 1f). Moreover, a higher percentage of intact mitochondria observed in the electron micrographs indicated preserved liver mitochondrial architecture in sericin-treated rats compared with that in non-treated rats (Fig. 1g–i).

### Proteomics results

**Figure 2:**
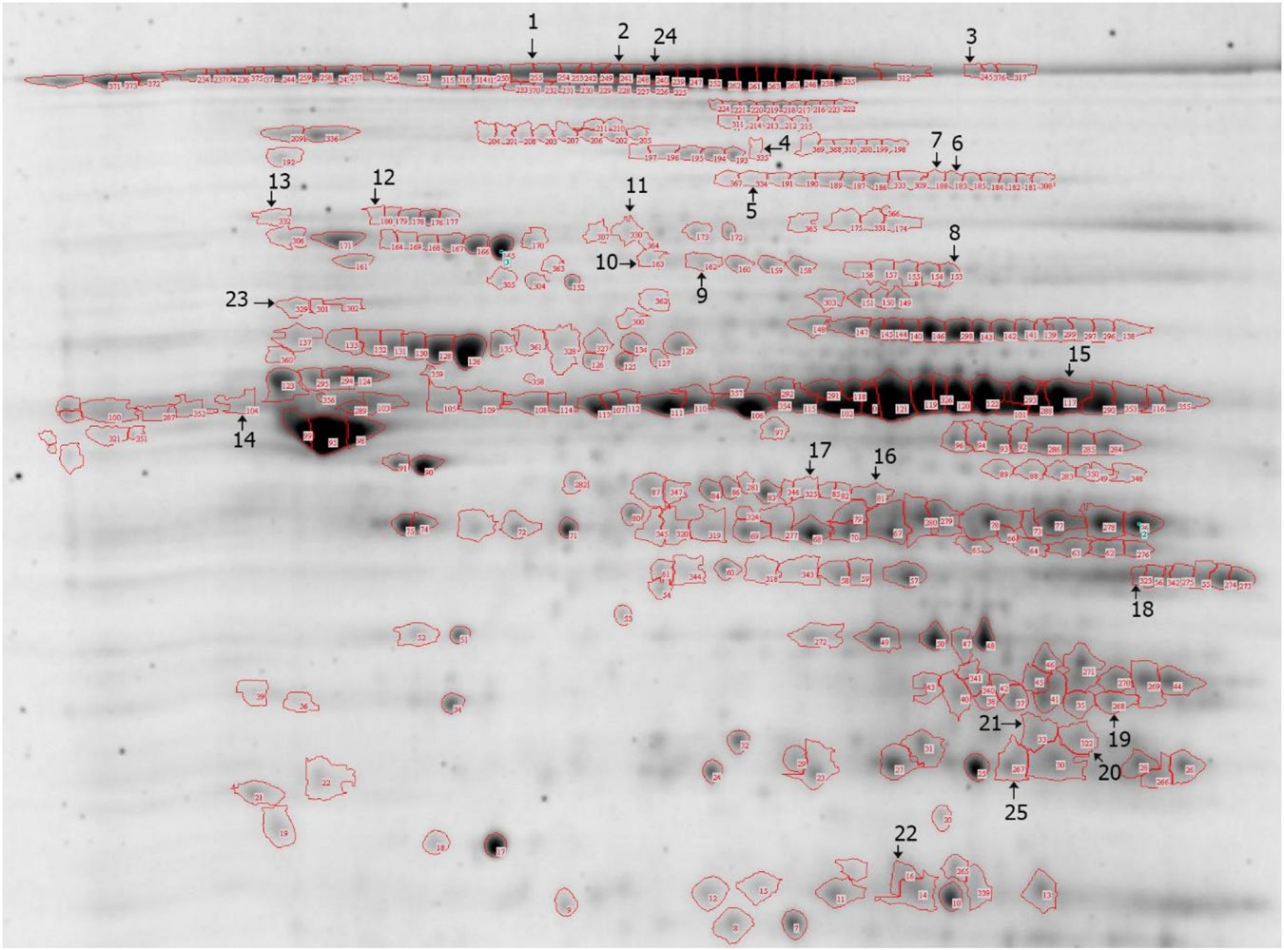
2DE image of the liver mitochondria extracted from non-treated rats. Proteins were separated using pH 3–10 IPG strips and SDS-PAGE and visualised by Flamingo fluorescent gel staining.

Overall, 376 proteins were separated by two-dimensional gel electrophoresis (2DE) analysis from three replicate sets of sericin-treated or non-treated liver mitochondrial extractions. Of these, 74 protein spots showed significantly different expression. Eight proteins were absent in sericin-treated rats. In addition, 26 proteins were upregulated in non-treated rats, whereas the rest were upregulated in sericin-treated rats. In this study, 25 differentially expressed proteins were selected for mass spectrometry (MS/MS) analysis and protein identification (Fig. 2). Up or downregulated proteins exhibiting in hepatic mitochondrial extraction was determined (Tables 1 and 2). From the selected protein spots, 80% (20/25) were mitochondrial proteins, whereas 20% (5/25) were contaminating proteins from the cytoskeleton and peroxisomes. The results revealed five categories of functional protein groups: (i) apoptotic [NADH-ubiquinone oxidoreductase 75 kDa subunit (NDUFS1)] and autophagic [mitochondrial elongation factor Tu (TUFM) and prohibitin-2 (PHB2)] proteins, (ii) energy maintenance proteins [haloacid dehalogenase-like hydrolase domain-containing protein 3 (HDHD3), succinate dehydrogenase (ubiquinone) flavoprotein subunit (SDHA), ATP synthase-α subunit precursor (ATPSA), enoyl-CoA hydratase domain-containing protein 3 (ECHDC3) and electron transfer flavoprotein subunit-α (ETFA)], (iii) anti-oxidative proteins [aconitate hydratase (ACO2) and Chain A, crystal structure of rat carnitine palmitoyltrasferase 2 (CPT-II)], (iv) liver-associated injury proteins [ornithine aminotransferase (OAT) and mitochondrial aspartate aminotransferase (GOT2), acyl-CoA synthase (ACSM1), hydroxyacyl-CoA dehydrogenase (HADH) and D-beta-hydroxybutyrate dehydrogenase (BDH1)] and (v) liver function improvement proteins [carbamoyl-phosphate synthase (ammonia) (CPS), aldehyde dehydrogenase (AD), uricase (UOX), dimethyl glycine dehydrogenase (DMGDH) and sarcosine dehydrogenase (SARDH)]. However, protein spots at numbers 6 and 7 were identical and were identified as ACO2. Non-categorised proteins [HSD17B4, keratin type 2 cytoskeletal cochleal, serum albumin precursor (ALB) and unnamed protein] were also indicated.

**Table 1:** Upregulated liver mitochondrial proteins in sericin-treated rats.

Spot no.	Intensity		ANOVA	Gene name	Protein name (name and source)	Number of peptide	Mw	pi	Sequence coverage	Function
	Non-treatment	Treatment								
6	0.0563781	0.111249	0.0036005	*Aco2*	Aconitate hydratase, mitochondria	21	85,380	7.87	45.0	Cell death, prevention of oxidative stress^22–24^
7	0.064487	0.117064	0.0357681	*Aco2*	Aconitate hydratase, mitochondria	20	85,380	7.87	46.2	
8	0.132741	0.252707	0.0000716	*CPT-II*	Chain A, Crystal Structure Of Rat Carnitine Palmitoyltransferase 2 In Space Group C2221, mitochondria	26	73,183	6.65	52.8	
9	0.0744941	0.211723	0.0008160	*Sdha*	Succinate dehydrogenase (ubiquinone) flavoprotein subunit, mitochondrial precursor (*Rattus norvegicus*), mitochondria	10	71,570	6.75	37.0	Contributes to energy synthesis via oxidative phosphorylation and Krebs cycle^20^
10	0.0384434	0.235219	0.0004450	*Alb*	Serum albumin precursor (*Rattus norvegicus*)	25	68,714	6.09	58.1	Maintains serum albumin level^54^
11	0.106439	0.200113	0.0246528	*Hdhd3*	Haloacid dehalogenase-like hydrolase domain-containing protein 3 (*Rattus norvegicus*), mitochondria	11	27,776	6.50	76.1	Contains hydrolase activity and preserves both metabolic and energy activity^18^
14	0.078924	0.492371	0.0256187	*ATPSa*	ATP synthase alpha subunit precursor (EC 3.6.1.3), partial (*Rattus norvegicus*), mitochondria	23	58,790	9.22	56.5	Energy trapping^16^
22	0.106439	0.200113	0.0246528	*Echdc3*	Enoyl-CoA hydratase domain-containing protein 3, mitochondrial precursor (*Rattus norvegicus*), mitochondria	10	32,365	8.83	51.3	Regulates fatty acid oxidation through beta-oxidative pathway^17^

**Table 2:** Downregulated liver mitochondrial proteins in sericin-treated rats.

Spot no.	Intensity		ANOVA	Gene name	Protein name (name and source)	Number of peptide	Mw	pi	Sequence coverage	Function
	Non-treatment	Treatment								
1	0.404	0.212	0.0000881	*Cps*	Carbamoyl-phosphate synthase (ammonia), mitochondria	54	164,476	6.33	52.9	Ammonia detoxification^35,36^
2	0.440614	0.085653	0.034025	—	Keratin type 2 cytoskeletal cochleal, cytoskeleton	57	57,574	6.11	33.2	Conserves the cytoskeleton^55^
3	0.175727	0.0695065	0.0197198	*Phb2*	Prohibitin-2 (*Rattus norvegicus*), mitochondria	10	33,292	9.83	41.8	Autophagy regulation^14,15^
4	0.035	n.d.	0.00378199	*Sardh*	Sarcosine dehydrogenase, mitochondria	19	101,376	6.15	63.7	Folate-binding protein^48,49^
5	0.0445298	n.d.	0.000586	*Dmgdh*	Dimethylglycine dehydrogenase, mitochondria	22	95,917	6.77	46.4	
12	0.062486	0.0256278	0.0457503	*Ndufs1*	NADH-ubiquinone oxidoreductase 75 kDa subunit, mitochondrial precursor (*Rattus norvegicus*), mitochondria	30	79,362	5.65	58.9	Specific caspase substrate during apoptosis^12^
13	0.0843622	n.d.	0.00577136	*Hsd17b4*	Peroxisomal multifunctional enzyme type II (*Rattus norvegicus*), peroxisome	36	79,364	8.69	61.1	Eliminates long-chain fatty acid^56^
15	1.99716	0.846697	0.0146911	*Aldh2*	Aldehyde dehydrogenase (*Rattus norvegicus*), mitochondria	15	55,566	6.69	47.3	Ethanol detoxification^40^
16	0.157365	0.081311	0.0489345	*Tufm*	Elongation factor Tu, mitochondrial precursor (*Rattus norvegicus*), mitochondria	22	49,491	7.23	73.2	Autophagy regulation^13^
17	0.109392	n.d.	0.000241	*Oat*	Ornithine aminotransferase, mitochondrial precursor (*Rattus norvegicus*), mitochondria	10	48,302	6.53	37.1	Induces apoptosis by enhanced cytosol protein degradation^26^
18	0.121606	n.d.	0.00517982	*Got2*	Aspartate aminotransferase, (*Rattus norvegicus*), mitochondria	18	47,284	9.13	57.2	Responsible for necrosis related pathology in the liver^27–29^
19	0.183181	0.0403365	0.00537057	*Uox*	Uricase (*Rattus norvegicus*), peroxisome	16	34,886	8.2	62.0	Regulates uric acid excretion^42^
20	0.182939	n.d.	0.0106031	*Hadh*	Hydroxyacyl-coenzyme A dehydrogenase, mitochondrial precursor (*Rattus norvegicus*), mitochondria	10	34,426	8.83	45.5	Enhances lipid deposition^33^
21	0.175727	0.0695065	0.0197198	*Bdh1*	D-beta-hydroxybutyrate dehydrogenase (*Rattus norvegicus*), mitochondria	7	38,322	8.93	25.6	Regulates ketone body biosynthesis^34^
23	0.0824859	n.d.	0.00367197	*ACSM1*	Acyl-coenzyme A synthetase ACSM1, mitochondrial (*Rattus norvegicus*), mitochondria	18	65,315	7.57	55.6	Involved in fatty acid biosynthesis^30–32^
24	0.451911	0.106341	0.0317921	—	Unnamed protein product (*Rattus norvegicus*)	15	61,389	8.05	45.7	—
25	0.401518	0.130023	0.0185507	*Etfa*	Electron transfer flavoprotein subunit alpha, (*Rattus norvegicus*), mitochondria	15	68,155	7.33	71.2	Plays a role in amino acid metabolism and beta-oxidation^19^

Our classification indicated that sericin regulated proteins involved in apoptosis, autophagy and energy maintenance (NDUFS1, TUFM, PHB2, OAT, GOT2, HDHD3, SDHA, ATPSA, ECHDC3 and ETFA); maintained its anti-oxidative property through ACO2 and CPT-II proteins; downregulated ACSM1, HADH and BDH1 proteins, leading to decreased liver pathology and promoted nitrogenous waste detoxification and other related liver functions (CPS, AD, UOX, DMGDH and SARDH).

### Immunoelectron microscopy and Western blot results

**Figure 3:**
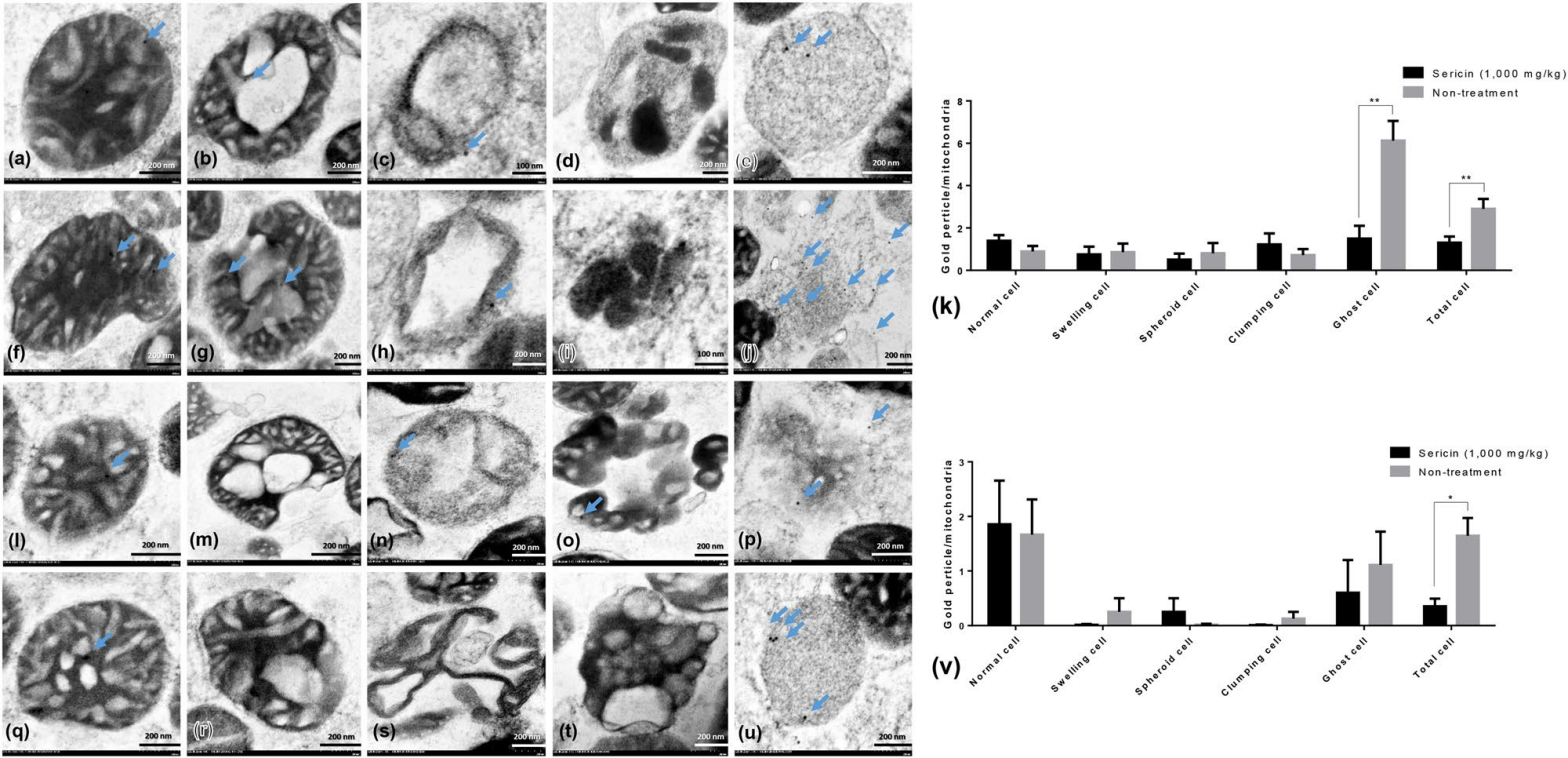
NDUFS1 and caspase-3 immunogold labelling at each stage of mitochondrial degeneration. NDUFS1 (**a**–**k**) and caspase-3 (**l**–**v**) gold labelling at all stages of liver mitochondrial degeneration: intact (**a**,**f**,**l** and **q**), swelling (**b,g,m** and **r**), spheroid (**c**,**h**,**n** and **s**), clumping (**d**,**i**,**o** and **t**) and ghost (**e**,**j**,**p** and **u**). Data in the bar graph is represented by mean ± SEM; *p < 0.05 and **p < 0.01.

Immunoelectron microscopy and Western blot were performed for validating the results of the proteomics analysis. Up- and downregulated mitochondrial proteins, NDUFS1, TUFM and HDHD3, were chosen for verifying their expression levels at each stage of liver mitochondrial degeneration fine morphology: intact (Fig. 3a,f,l and q), swelling (Fig. 3b,g,m and r), spheroid (Fig. 3c,h,n and r), clumping (Fig. 3d,i,o and t) and necrosis or ghost (Fig. 3e,j,p and u) mitochondria, as described previously^1^. Swelling mitochondria were represented by singular or multiple distensions in the intercellular matrix in association with cristae loss and intact double membranes. Mitochondrial spheroidicity was categorised by a high level of swelling with severe distension of the intercellular matrix, formation of multiple cysts and loss of double membranes. Clumping mitochondria were indicated by a complete loss of cristae and double membranes accompanied by electron-dense material clumping of the degenerative or fragment mitochondria. Lastly, ghost mitochondria were the final step of mitochondrial alterations in which the remaining mitochondrial fragments presented as a fine granular substance of electron-dense material with or without its boundary.

**Figure 4:**
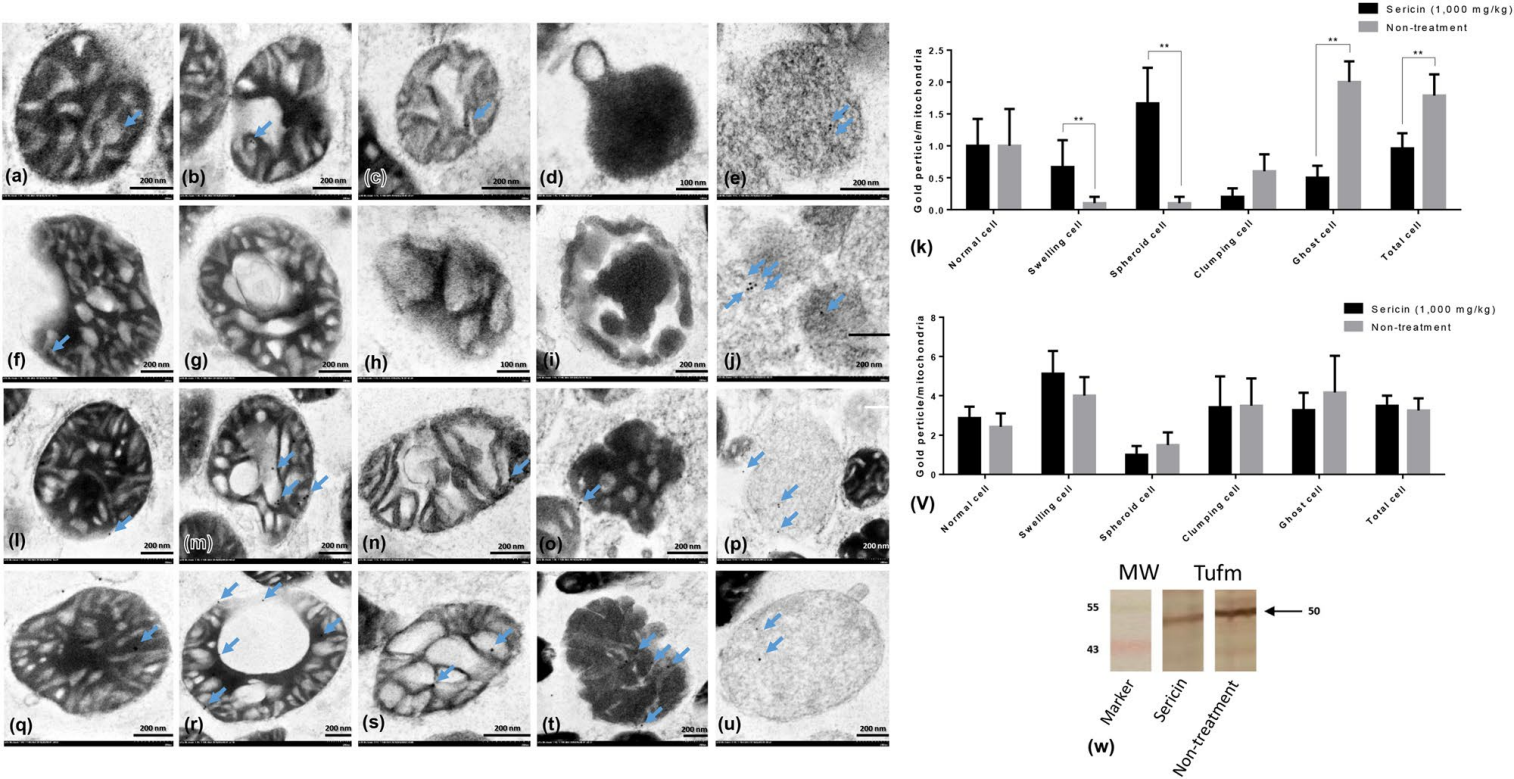
TUFM and LC3 immunogold labelling at each stage of mitochondrial degeneration and Western blot analysis of TUFM. TUFM (**a**–**k**) and LC3 (**l**–**v**) gold labelling at all stages of liver mitochondrial degeneration: intact **(a**,**f**,**l** and **q**), swelling (**b**,**g**,**m** and **r**), spheroid (**c**,**h**,**n** and **s**), clumping (**d**,**i**,**o** and **t**) and ghost (**e**,**j**,**p** and **u**). Data in the bar graph is represented by mean ± SEM; **p < 0.01. Western blot analysis of the TUFM level between treated and non-treated rats (w). Full Western blot was provided in the supplementary file.

**Figure 5:**
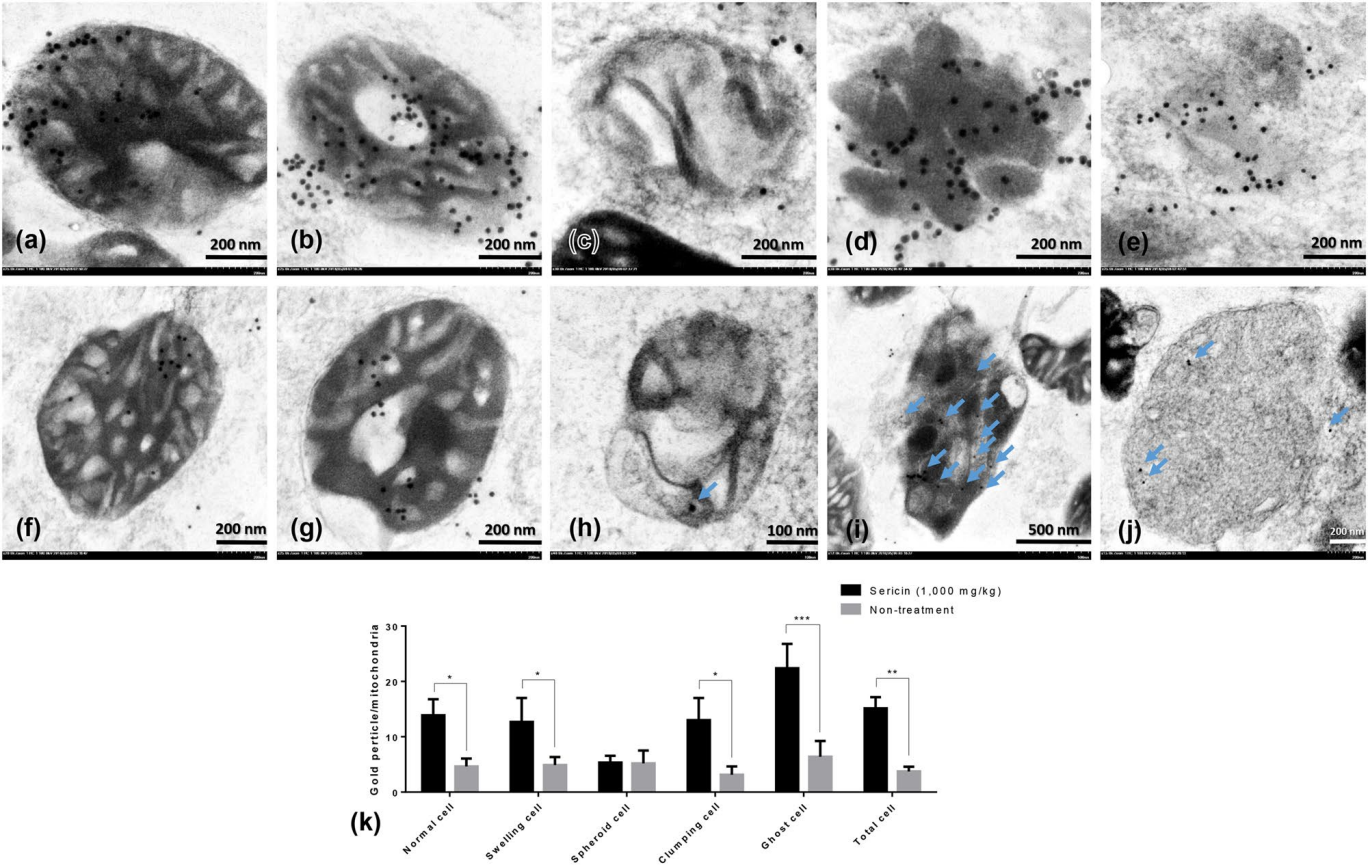
HDHD3 immunogold labelling at each stage of mitochondrial degeneration. HDHD3 (**a–k**) gold labelling at all stages of liver mitochondrial degeneration: intact (**a,f**), swelling (**b,g**), spheroid (**c,h**), clumping (**d,i**) and ghost (**e,j**). Data in the bar graph is represented by mean ± SEM; *p < 0.05 and **p < 0.01.

In addition, to relate the function of these selected proteins to their roles in the pathogenesis of hepatic steatosis and dysmorphic mitochondrial under conditions of hypercholesterolaemia, the expression of apoptotic protein (caspase-3), mitochondrial fission protein (DRP1), mitochondrial fusion protein (OPA1) and autopagic protein (LC3) was examined. The results indicated that the expression of NDUFS (Fig. 3a–k) and TUFM (Fig. 4a–k) in the hepatic mitochondria from sericin-treated rats was significantly lower than that the hepatic mitochondria from non-treated rats, particularly in ghost cells. Conversely, the expression of HDHD3 in sericin-treated rats was significantly upregulated in most stages of mitochondrial alterations compared with that in non-treated rats (Fig. 5a–k). In addition, Western blot showed a high TUFM protein level in non-treated rat compared with that in sericin-treated rat (Fig. 4w). Therefore, the protein expression level determined by gold labelling was in agreement with the results from our proteomics analysis, thereby reflecting consistency in our proteomics results.

**Figure 6:**
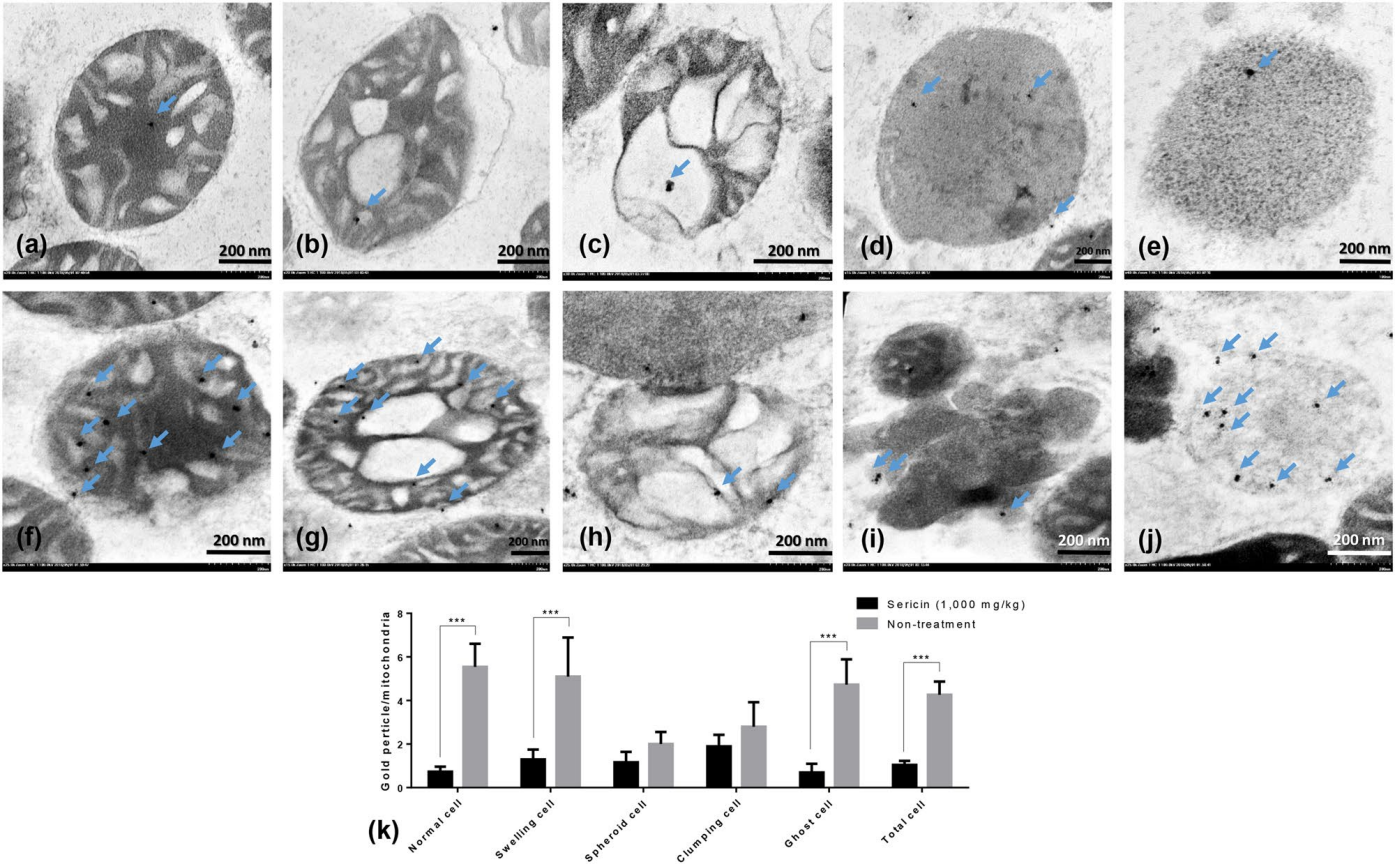
DRP1 immunogold labelling at each stage of mitochondrial degeneration. DRP1 (**a**–**k**) gold labelling at all stages of liver mitochondrial degeneration: intact (**a**,**f**), swelling (**b**,**g**), spheroid (**c,h**), clumping (**d,i**) and ghost (**e,j**). Data in the bar graph is represented by mean ± SEM; ***p < 0.001.

**Figure 7:**
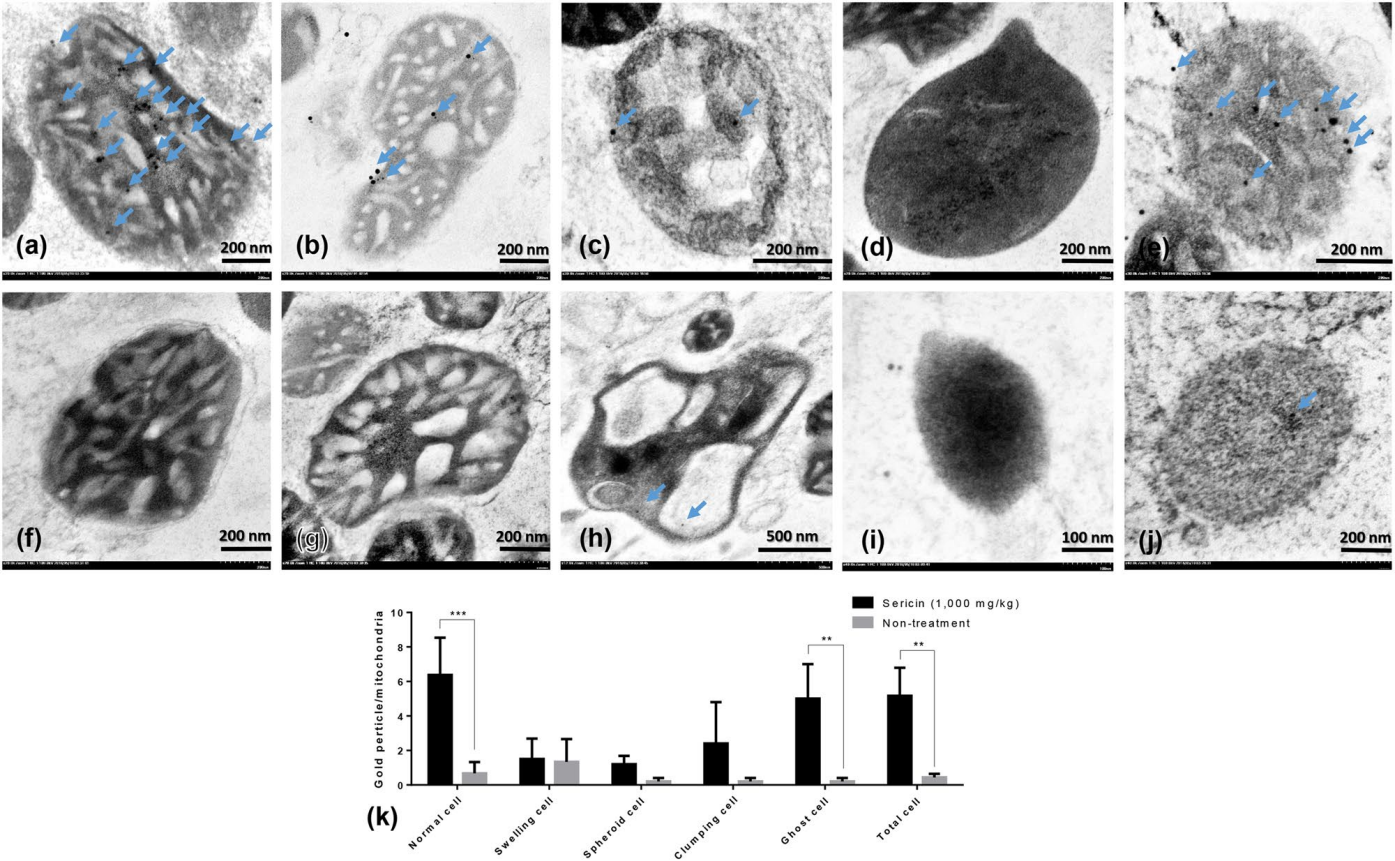
OPA1 immunogold labelling at each stage of mitochondrial degeneration. OPA1 (**a–k**) gold labelling at all stages of the liver mitochondria: intact (**a,f**), swelling (**b,g**), spheroid (**c,h**), clumping (**d**,**i**) and ghost (**e**,**j**). Data in the bar graph is represented by mean ± SEM; **p < 0.01 and ***p < 0.001.

**Figure 8:**
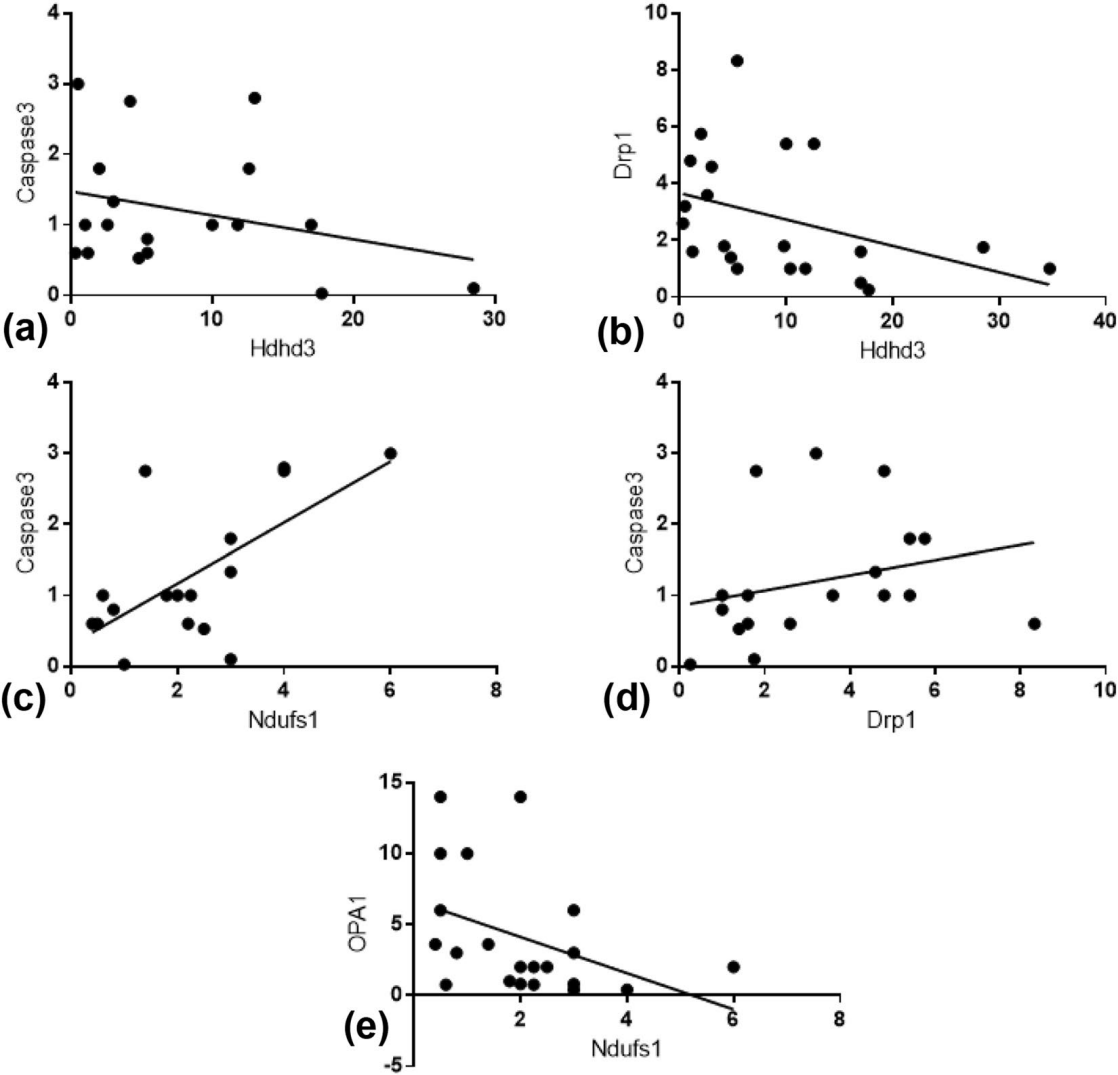
Spearman’s correlation among selected mitochondrial proteins. Positive and negative correlations are demonstrated (**a–e**).

Caspase-3 (Fig. 3l–v) and LC3 (Fig. 4l–v) gold labelling was decreased in ghost mitochondria from sericin-treated rats compared with that in ghost mitochondria from non-treated rats. Considering fission and fusion phenomena, dynamic processes involved in mitochondria homeostasis and DRP1 and OPA1 gold labelling were evaluated in the present study. The results revealed that compared with non-treated rats, almost all mitochondrial stages in sericin-treated rats had lower expression of DRP1 (Fig. 6a–k). In contrast to the expression of OPA1, the total expression in sericin-treated rats was significantly increased compared with that in non-treated rats, particularly in intact and ghost mitochondria (Fig. 7a–k). The present study also revealed correlations contributing to the role of sericin in mitochondrial alterations under high blood cholesterol levels using Spearman’s rho correlation test. Low-energy mitochondria had an increased apoptotic and fission protein labelling levels, as characterised by a negative correlation between HDHD3 and caspase-3 (r = −0.456, p = 0.029) and between HDHD3 and DRP1 (r = −0.554, p = 0.006) (Fig. 8a–b). A positive correlation was also found between DRP1 and caspase-3 (r = 0.423, p = 0.044) (Fig. 8d). Finally, although NDUFS1 showed a positive correlation with caspase-3 (r = 0.558, p = 0.006), a negative correlation was observed between NDUFS1 and OPA1 (r = −0.546, p = 0.007) (Fig. 8c,e).

## Discussion

In this study, we used proteomics for characterising and comparing liver mitochondrial proteins from sericin-treated and non-treated rats with hypercholesterolaemia. The highly significant proteins were categorised into five groups depending on their functions and association with the pathogenesis of steatosis: (i) apoptosis and autophagy, (ii) energy maintenance, (iii) anti-oxidative stress, (iv) liver injury (e.g. fat deposition) and (v) liver function improvement (e.g. nitrogen waste detoxicfication and folate-binding protein). These findings are consistent with those of our recent study, which reported that lipid dysregulation leads to mitochondrial alteration and dysfunction and high apoptosis levels in association with defective energy synthesis^1^.

Apoptosis contributes to different stages of mitochondrial biosynthesis, fission (apoptotic induction process) or fusion (maintenance of mitochondrial function) in relation to dysmorphic mitochondria: (i) swelling, (ii) spheroidicity, (iii) collapse or clumping and (iv) necrosis^1^. During apoptosis, disturbed mitochondrial function is mediated by mitochondrial NDUFS1, a critical caspase substrate in the mitochondria^12^. Cleavage of NDUFS1 by caspase activation due to permeabilisation of the outer mitochondrial membrane and cytochrome release is mainly associated with apoptosis. High NDUFS1 levels were found in the liver mitochondria from non-treated rats. Our proteomics results also showed that an increase in the autophagy regulation proteins TUFM and PHB2 was observed in non-treated rats. In agreement with the results of gold labelling, TUFM was higher in the ghost mitochondria of non-treated rats than in that of sericin-treated rats. High expression of TUFM induces autophagy^13^. PHB2 is an inner mitochondrial membrane mitophagy receptor that is imperative for targeting mitochondria for autophagic degradation^14^. This may be associated with the high prevalence of dysmorphic mitochondria in non-treated rats due to the central role autophagy plays in mitochondrial homeostasis to remove unwanted or damaged mitochondria^15^. Unfortunately, instead of upregulated LC3 in the mitochondria of non-treated rats, there was no difference in LC3 gold labelling between sericin-treated and non-treated rats.

The main function of mitochondria is being the powerhouse of the cell. This study provided evidence of high expression of some mitochondrial energy maintenance proteins in sericin-treated rats, such as HDHD3, SDHA, ATPSA, ECHDC3 and ETFA^16–20^. In addition, it is well known that impairment of fatty acid oxidation or energy production in the mitochondria is significantly associated with the pathogenesis of non-alcoholic steatohepatitis (NASH)^21^. This indicates that sericin alleviates the severity of hepatic steatosis by preserving the generation of mitochondrial energy and improves the mitochondrial architecture by modulating DRP1 and caspase-3 activity.

The present study demonstrated that sericin induces an antioxidative environment characterised by upregulation of several mitochondrial proteins, such as mitochondrial ACO2 and CPT-II. ACO2 is a catalytic enzyme in the tricarboxylic acid cycle^22^, which plays a role in oxidative stress tolerance, leading to cell death prevention^23^, whereas CPT-II controls the long-chain fatty acid beta-oxidation level, thereby contributing to appropriate oxidative stress and inflammatory inductions^24,25^. In agreement with our previous report indicating anti-oxidative properties of sericin in the heart and liver mitochondria, as shown by oxidative stress markers (e.g. superoxide dismutase, MDA and NRF-2)^1,2^, the present proteomics study confirmed that sericin preserves liver mitochondria from oxidative stress by maintaining ACO2 and CPT-II functions.

Moreover, abnormal mitochondria cause an accumulation of OAT, leading to cytosolic protein degradation^26^. In agreement with liver damage-related protein, GOT2, a transamination catalyst for kynurenic acid formation, is responsible for metabolic exchange between the mitochondria and cytosol^27^. Elevated GOT2 levels may be associated with some pathologies in the liver, particularly ischaemic necrosis^28^ and liver injuries^29^. The present study indicated that in high-cholesterol-fed rats, a decrease in hepatic injury from the hypercholesterolaemic stage might be attributable to the absence of GOT2.

Steatosis, a hepatocytic fatty change, is an imbalanced fat metabolism in the liver characterised by over-accumulation of triglyceride fat, reflecting impairment in synthesis or elimination of lipids. The present study demonstrated that sericin modulates mitochondrial ACSM1, which plays a major role in fatty acid biosynthesis^30^. Overexpression of ACSM1 results in the deposition of large amounts of triglycerides in hepatocytes^31^ and an increase in free fatty acid transportation into the cell^32^. The presence of ACSM1 in non-treated rats indicated high deposition of triglycerides in hepatocytes and led to higher severity of steatosis than that in sericin-treated rats. Moreover, mitochondrial HADH, a mitochondrial protein that enhances lipid deposition^33^, was significantly expressed in non-treated rats. In addition, BDH1 was significantly higher in non-treated rats than in sericin-treated rats. BDH1 plays a role in the regulation of ketone body biosynthesis, particularly in adipose tissue development^34^. High BDH1 levels may induce lipid deposition, as demonstrated in non-treated rats.

Mitochondrial CPS, a catalytic enzyme in the urea cycle, is an important modulator of ammonia detoxification^35,36^ that is activated by *N*-acetyl-L-glutamate, a first step activator of the urea cycle in mitochondria^37^. High production of CPS is responsible for high ammonia levels and leads to increased urea synthesis. Disruption of the urea cycle can cause steatosis and focal hepatic necrosis^38^. Increases or decreases in the levels of enzymes essential for the mitochondrial urea cycle reflect mitochondrial impairment in NASH and steatosis^39^. Our results suggest that sericin balances urea synthesis in high-cholesterol-fed rats as high CPS levels were observed in non-treated rats in association with the high severity of hepatic steatosis.

Mitochondrial AD plays a vital role in the detoxification of the ethanol metabolite, acetaldehyde^40^. High AD levels seem to be beneficial to ethanol intoxication. However, a significant difference in AD activity is not frequently observed between alcoholic and non-alcoholic liver, but its activity is highly altered following liver damage^41^. Non-treated rats showed high AD levels compared with sericin-treated rats, which possibly contributed to the high severity of hepatic steatosis in relation to the presence of some toxic metabolites.

Mitochondrial UOX, a homotetrameric enzyme, converts uric acid to allantoin, which is a more soluble, simple form for excretion^42^. Endogenous uric acid is mainly produced in the liver^43^. High blood cholesterol level is closely related to uric acid levels^44^ under oxidative stress conditions^45^. Non-treated rats had higher uricase levels than sericin-treated rats. This indicated that non-treated rats had high uric acid levels that may lead to hepatic steatosis and an enhanced oxidative stress environment.

There is strong evidence to show that folate depletion is usually found in non-alcoholic fatty liver or other conditions of liver damage because of perturbation of hepatic metabolism^46,47^. Mitochondrial SARDH and DMGDH are the major folate-binding proteins that contribute to sarcosine metabolism^48^. Increased SARDH causes folate deficiency^49^. Therefore, folate depletion may have caused the presence of SARDH and DMGDH in non-treated rats but not in the sericin-treated group.

In summary, the mitochondrial ultrastructure in the liver was improved by sericin activity under hypercholesterolaemic conditions. Dysmorphic mitochondria were decreased in sericin-treated rats because of sericin’s modulatory effects on apoptotic (NDUFS1) and autophagic (TUFM and PHB2) mitochondrial proteins; this is in agreement with the caspase-3, OPA1 and DRP1 levels in the hepatic mitochondria. Sericin not only maintains energy production through enhancement of HDHD3, SDHA, ATPSA, ECHDC3 and ETFA function but also demonstrates anti-oxidative activity via ACO2 and CPT-II proteins in the hepatocytes. Our proteomics study also revealed that lipid deposition and hepatocyte injury in the liver were decreased in association with the expression of OAT, GOT2, ACSM1, HADH and BDH1 proteins. These mechanisms ameliorated hepatic steatosis and improved other liver functions, characterised by expression of CPS, AD, UOX, SARDH and DMGDH. Our findings may contribute to the understanding of mechanisms by which sericin recovers hypercholesterolaemia, based on liver mitochondria conformation and hepatic steatosis, and highlights sericin as a leading candidate and as a blood cholesterol-lowering food additive.

## Materials and Methods

### Ethics statement

This study was approved by the Animal Care and Use Committee, Faculty of Medicine, Chulalongkorn University (Approval No. 16/2558). All animal studies were performed in accordance with the Animal for Scientific Purposes Act, B.E. 2558 (A.D. 2015), Thailand.

### Sericin extraction

Silkworm (*Bombyx mori*) cocoons were obtained from Chul Thai Silk Co., Ltd. (Petchaboon Province, Thailand). Sericin was extracted by heat induction to achieve appropriate purity amino acids, using methods described previously^50^. After autoclaving the fresh cocoon shells in distilled water at 120 °C for 60 min, the supernatant was filtered, frozen and lyophilised.

### Animal experiment protocol

#### Induction of cholesterolaemia and sericin treatment

Eight-week-old female Wistar rats (200–300 g) were obtained from the National Laboratory Animal Center, Mahidol University (NLAC-MU). To induce hypercholesterolaemia, they were fed 6% cholesterol-coated diets (Perfect Companion Ltd., Thailand) *ad libitum* for 6 weeks prior to the experiment^1^. Next, rats were separated into two groups according to the treatment, i.e. sericin-treated and non-treated rats (six rats per group), and were administered 1000 mg/kg of sericin or distilled water, respectively, by oral gavage for 4 weeks. All rats were humanely sacrificed by an overdose of isoflurane inhalation.

#### Specimen collection

Blood specimens were collected by cardiac puncture and centrifuged at 1500 × *g* for 15 min. Serum (six samples/group) was separated; blood lipid (cholesterol and HDL) profiles were determined by the Quality Control Division, NLAC-MU. To exclude technical artefacts from mitochondrial architecture studies, the specimen must be maintained under ice-cold condition throughout the experiment. Liver specimens were immediately collected and stored in ice-cold homogenate buffer (0.32 M sucrose, 1 mM EDTA, and 10 mM Tris-HCl; pH 7.4) when performing a standard protocol for mitochondrial extraction, as described previously^1,2^. Liver specimens were also collected and separated for histopathology and electron microscopy analysis and preserved in 10% neutral buffer formalin for 48 h and in 2.5% glutaraldehyde in 0.1 M sucrose phosphate buffer (SPB), pH 7.4, respectively.

### Mitochondrial extraction

After specimen collection, mitochondrial extraction was performed within 1–2 h to prevent cellular damage. Liver specimens from each group were pooled (six livers/group), weighed (15.24 g of liver from sericin-treated rats and 13.75 g of liver from non-treated rats), chopped and washed in homogenate buffer. Liver specimens were homogenised in a glass Potter–Elvehjem tissue grinder with an appropriate volume of the homogenate buffer (4 mL of homogenate buffer/1 g of liver specimen). During this step, several up and down strokes were performed using a motor-driven Teflon pestle at 600 rpm. Next, homogenates were centrifuged at 1000 × *g* at 4 °C for 5 min. The supernatants were collected and centrifuged at 15,000 × *g* at 4 °C for 2 min. The mitochondrial pellets were collected and washed several times in homogenate buffer. The pellets were resuspended in ice-cold final equilibrated buffer [250 mM sucrose, 5 mM KH_2_PO_4_, 10 mM Tris-HCl, and 2 mg/mL bovine serum albumin (BSA); pH 7.2], and 200 μL of resuspended pellet was fixed in 2.5% glutaraldehyde in 0.1 M SPB for electron microscopy analysis. The mitochondrial protein content was measured by protein assay (Bio-Rad^®^) using a spectrophotometer (NanoDrop-1000, Thermo Scientific).

### Proteomic studies

#### Two-dimensional gel electrophoresis

Each mitochondrial protein was loaded onto Immobiline^™^ Drystrip immobilised pH gradient (IPG) strips (13 cm, pH 3–10 NL, GE healthcare^®^) using the passive rehydration method for 12 h. Isoelectric focussing was performed using Ettan IPGphor (GE healthcare^®^) at 20 °C, and the gel strips were equilibrated with the equilibration buffer twice for 15 min each, as described previously^51^. The second dimension run was performed as follows. Each equilibrated gel strip was loaded onto a 12.5% sodium dodecyl sulphate polyacrylamide gel, sealed with the agarose sealing solution and supplied with current, using a vertical slab gel electrophoresis unit (SE 600 Chroma Hoefer^®^), of 15 mA/gel for 30 min and then with a current of 30 mA/gel until the bromophenol blue reached the bottom of the gels. Gels were fixed with 10% acetic acid in 40% ethanol for 2 h, stained with the Flamingo^™^ Fluorescent gel stain (Bio-Rad^®^) for 18 h and washed with distilled water. 2DE was performed in triplicate.

#### Image analysis

Gels were scanned using a Typhoon Trio Variable Mode Imager (GE Healthcare^®^) under a 532-nm green laser and 555-nm long pass emission filter. Image Master 2D software (GE healthcare^®^) was used for detecting, quantifying and comparing the spots depending up on their OD values. Spots were considered to be significant at p < 0.05 using analysis of variance (ANOVA). Twenty-five significant spots were chosen for protein identification.

#### In-gel tryptic digestion

Selected spots (Fig. 1) were collected, dehydrated in acetonitrile (HPLC grade, Merk^®^), reduced in dithiothreitol DTT solution (10 mM DTT in 100 mM NH_4_HCO_3_) for 1 h at 56 °C and alkylated in iodoacetamide (55 mM iodoacetamide in 100 mM NH_4_HCO_3_) for 45 min at room temperature with light protection. Spotted gels were washed using 100 mM NH_4_HCO_3_ and digested in trypsin digestion buffer [12.5 ng/µL of trypsin (Promega) in 50 mM NH_4_HCO_3_] for 45 min at 4 °C. Excess trypsin was removed by incubating the gels in 50 mM NH_4_HCO_3_ buffer overnight at 37 °C. Tryptic digests were performed using 20 mM NH_4_HCO_3_ buffer and 5% formic acid in 50% acetonitrile for 20 min at room temperature. Finally, specimens were centrifuged at 14,000 × *g* for 1 min. The supernatants were collected and dried in SpeedVac concentrator (Thermo Scientific) at room temperature.

#### Mass spectrometry analysis and protein identification

MS/MS analysis was performed using Nano LC/MS/MS, Maxis UHR-QTOF (Bruker) coupled to a Nano LC System (Dionex). DataAnalysis software, version 3.4, was used for converting LC-MS/MS files to mascot generic files, and Mascot version 2.4.1 (Matrix Science, London, UK) was used for identifying the mitochondrial proteins.

### Morphological studies

#### Histopathology analysis

Histopathology was used for measuring the severity of hepatic steatosis from hypercholesterolaemia. Fixed liver specimens (six livers/group) were used for standard tissue processing and stained with haematoxylin and eosin. The severity of hepatic steatosis between sericin-treated and non-treated rats was examined under a light microscope, and the percentage of fat deposition/high-power field was quantified using image J programme (NIH, USA), as described previously^1,2,51–53^. Briefly, colour images (10 images/liver) were acquired using light microscope (BX41, Olympus^®^, Japan) and digital camera (DP20, Olympus^®^, Japan) at 400× magnification. The images were then transformed to a grey scale. The fat deposit area was localised and measured into a percentage area fraction using threshold adjustment.

#### Conventional electron microscopy

Electron microscopy was used for examining the ultrastructure of hepatic steatosis. Secondary fixation of the six liver specimens from each group was performed using 1% osmium tetroxide, dehydrated in graded ethanol, infiltrated in a series of LR white resin (EMS^®^, USA), embedded in pure LR white (EMS^®^, USA), polymerised at 60 °C for 48 h, cut into 100-nm-thick sections and stained with lead citrate and uranyl acetate. The liver ultrastructure was examined under transmission electron microscope (model HT7700, Hitashi, Japan). Fat droplets in hepatocytes were counted (50 cells per group) and compared between sericin-treated and non-treated rats.

#### Immunogold labelling technique

Immunogold labelling was performed for confirming and validating the proteomics study results on pathogenesis of hypercholesterolaemia-induced hepatic steatosis and dysmorphic mitochondria. In this study, several markers (MyBioSource, USA) were used as primary antibodies: apoptotic markers, mouse polyclonal NDUFS1 and caspase-3; autophagic markers, mouse polyclonal TUFM and rabbit polyclonal anti-LC3; fusion and fission markers, rabbit polyclonal OPA1 and DRP1 and energy marker, rabbit polyclonal anti-HDHD3.

The mitochondrial pellet from the pooled liver extract in each group was secondary fixed, and tissue processing was performed as described previously. Sections were blocked using 50 mM glycine in phosphate-buffered saline (PBS) followed by 5% BSA (EMS, USA) in PBS for 30 min each. The sections were incubated with 1:50 diluted primary antibodies for 1 h prior to applying goat anti-rabbit or mouse IgG conjugated with ultra-small (3–5 nm) or 10-nm gold particles (EMS^®^, USA). Between each step, sections were washed using 0.1% BSA in PBS several times. To improve the contrast of the gold particle labelling, a silver enhancement kit (Aurion R-Gent SE-EM kit, EMS, USA) was used after rigorously washing the sections with distilled water. Finally, the sections were stained with lead citrate and uranyl acetate prior to transmission electron microscope examination. The number of labelled gold particles was counted for each ultra-characteristic stage of liver mitochondrial degeneration: intact, swelling, spheroidicity, collapse or clumping and necrosis. At least 100 mitochondria (20 mitochondria/stage) were evaluated per group.

### Western blot analysis

To confirm the proteomic results, Western blot was used for determining the level of one selected marker, TUFM. Fifty micrograms of mitochondrial protein from treated and non-treated rats was loaded onto 10% sodium dodecyl sulphate polyacrylamide gel electrophoresis and transferred to a transfer membrane. The membrane was blocked with 10% skin milk, incubated in mouse polyclonal anti-mitochondrial TUFM overnight and incubated in HRP-conjugated secondary antibody for 1 h. The signal was visualised using a DAB chromogen.

### Statistics

Statistical analysis was performed using GraphPad Prism^®^ version 5. Differences between quantitative parameters were compared using Student’s *t*-test and presented by mean ± SEM. Bivariate correlation was examined using Spearman’s test. The level of statistical significance was set at p < 0.05.

## Electronic supplementary material

Supplementary information

## Data Availability

Datasets generated and/or analysed during the current study are available from the corresponding author upon request.
