# Author Correction: Adaptive effect of sericin on hepatic mitochondrial conformation through its regulation of apoptosis, autophagy and energy maintenance: a proteomics approach

**DOI:** 10.1038/s41598-023-31599-4

**Published:** 2023-03-22

**Authors:** Sumate Ampawong, Duangnate Isarangkul, Onrapak Reamtong, Pornanong Aramwit

**Affiliations:** 1 Department of Tropical Pathology, Faculty of Tropical Medicine, Mahidol University, Ratchawithi Road, Ratchathewi, Bangkok, 10400 Thailand; 2 Department of Microbiology, Faculty of Science, Mahidol University, 272, Rama VI Road, Ratchathewi, Bangkok, 10400 Thailand; 3 Department of Molecular Tropical Medicine and Genetic, Faculty of Tropical Medicine, Mahidol University, Ratchawithi Road, Ratchathewi, Bangkok, 10400 Thailand; 4 Bioactive Resources for Innovative Clinical Applications Research Unit and Department of Pharmacy Practice, Faculty of Pharmaceutical Sciences, Chulalongkorn University, PhayaThai Road, Phatumwan, Bangkok, 10330 Thailand

Correction to: *Scientific Reports* 10.1038/s41598-018-33372-4, published online 08 October 2018

This Article contains errors in Figures 3 and 4, where the electron micrographs regarding an overlapped area of these images to another are incorrect in labels (q), (o), and (p). The correct Figures 3 and 4 and accompanying legends appear below.

**Figure 3:**
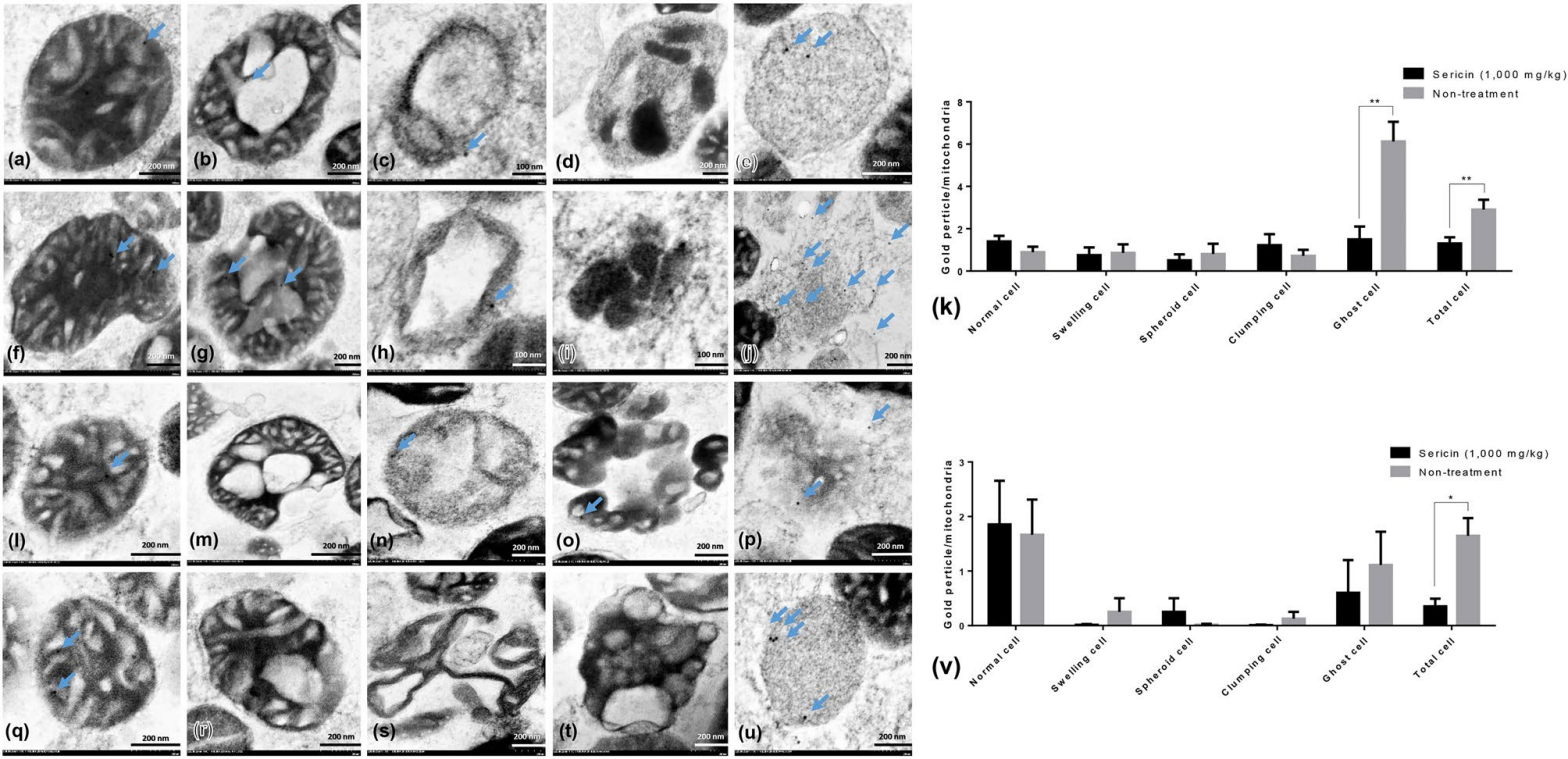
NDUFS1 and caspase-3 immunogold labelling at each stage of mitochondrial degeneration. NDUFS1 (**a**–**k**) and caspase-3 (**l**–**v**) gold labelling at all stages of liver mitochondrial degeneration: intact (**a**,**f**,**l** and **q**), swelling (**b,g,m** and **r**), spheroid (**c**,**h**,**n** and **s**), clumping (**d**,**i**,**o** and **t**) and ghost (**e**,**j**,**p** and **u**). Data in the bar graph is represented by mean ± SEM; *p < 0.05 and **p < 0.01.

**Figure 4:**
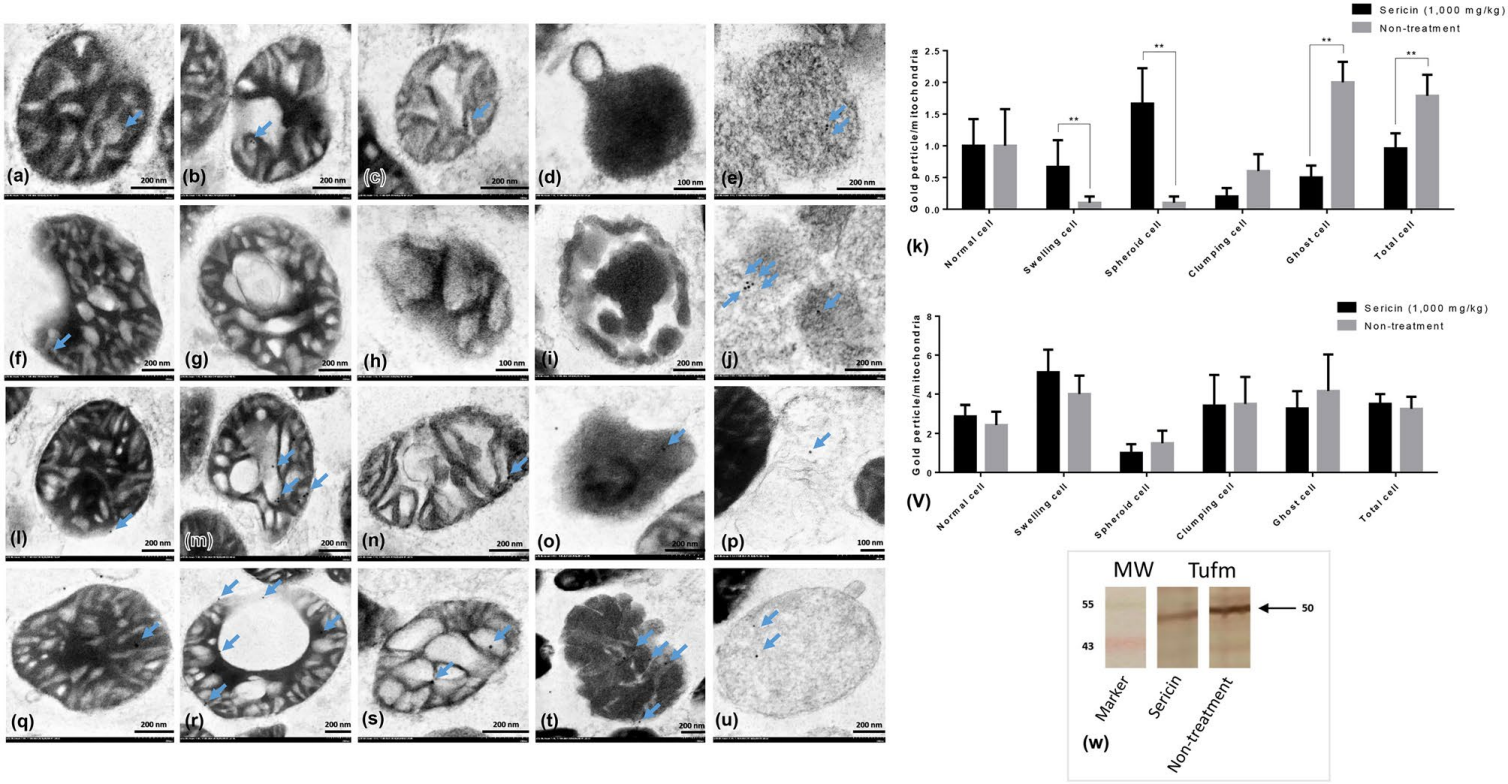
TUFM and LC3 immunogold labelling at each stage of mitochondrial degeneration and Western blot analysis of TUFM. TUFM (**a**–**k**) and LC3 (**l**–**v**) gold labelling at all stages of liver mitochondrial degeneration: intact (**a**,**f**,**l** and **q**), swelling (**b**,**g**,**m** and **r**), spheroid (**c**,**h**,**n** and **s**), clumping (**d**,**i**,**o** and **t**) and ghost (**e**,**j**,**p** and **u**). Data in the bar graph is represented by mean ± SEM; **p < 0.01. Western blot analysis of the TUFM level between treated and non-treated rats (w). Full Western blot was provided in the supplementary file.

