# Supplementary material for: Adaptive effect of sericin on hepatic mitochondrial conformation through its regulation of apoptosis, autophagy and energy maintenance: a proteomics approach
Source: Sci Rep. 2018 Oct 8;8:14943. doi: 10.1038/s41598-018-33372-4 (PMC6175853; doi:10.1038/s41598-018-33372-4)

**Title: Adaptive effect of sericin on hepatic mitochondrial conformation through its regulation of apoptosis, autophagy and energy maintenance: a proteomics approach**

Sumate Ampawong^1^, Duangnate Isarangkul^2^, Onrapak Reamtong^3^, Pornanong Aramwit^4,*^

^1^Department of Tropical Pathology, Faculty of Tropical Medicine, Mahidol University, Ratchawithi Road, Ratchathewi, Bangkok 10400 Thailand; Tel: (+662) 3549100; Fax: (+662) 6447938; E-mail address: [am_sumate@hotmail.com](mailto:am_sumate@hotmail.com)

^2^Department of Microbiology, Faculty of Science, Mahidol University,

272, Rama VI Road, Ratchathewi, Bangkok, 10400, Thailand; Tel: (+662) 2015671; Fax: (+662) 6445411; E-mail address: duangnate.int@mahidol.ac.th

^3^Department of Molecular Tropical Medicine and Genetic, Faculty of Tropical Medicine, Mahidol University, Ratchawithi Road, Ratchathewi, Bangkok 10400 Thailand; Tel: (+662) 3069138; Fax: (+662) 3069139; E-mail address: [Onrapak.lea@](mailto:Onrapak.lea@)mahidol.ac.th

^4^Bioactive Resources for Innovative Clinical Applications Research Unit and Department of Pharmacy Practice, Faculty of Pharmaceutical Sciences, Chulalongkorn University, PhayaThai Road, Phatumwan, Bangkok 10330 Thailand; Tel: (+662) 2188403; E-mail address: [aramwit@gmail.com](mailto:aramwit@gmail.com)

***Corresponding author**: Prof. Dr. Pornanong Aramwit


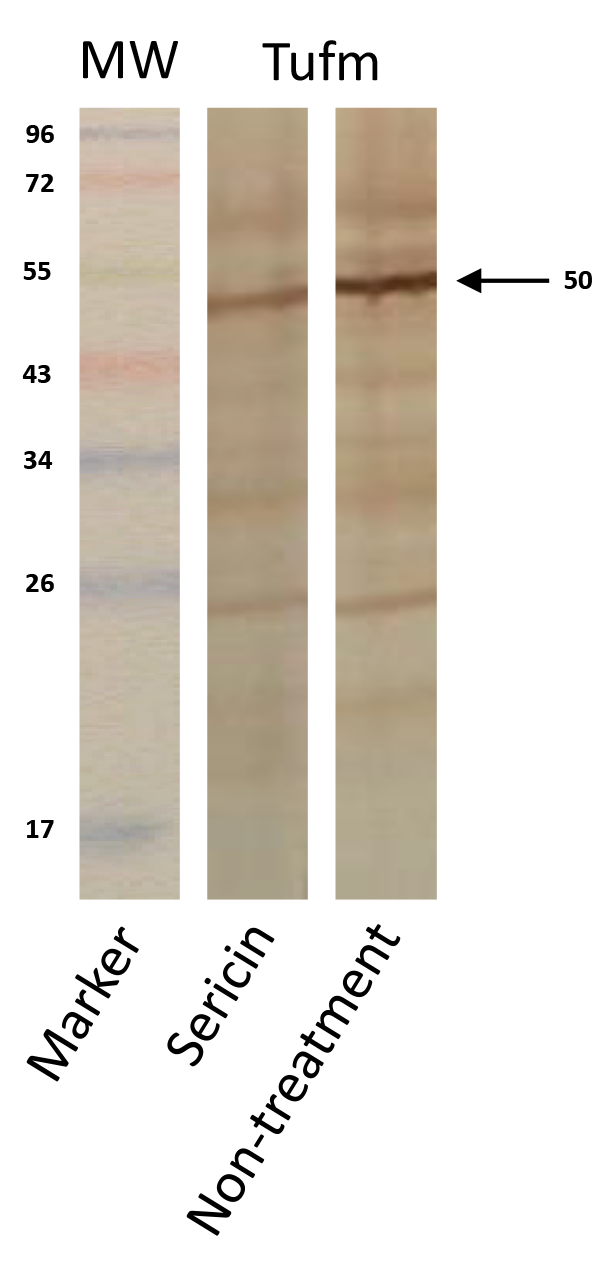

Supplement: Supplementary file 1 — Supplementary information [file 41598_2018_33372_MOESM1_ESM.docx]
